# TIR8/SIGIRR is an Interleukin-1 Receptor/Toll Like Receptor Family Member with Regulatory Functions in Inflammation and Immunity

**DOI:** 10.3389/fimmu.2012.00322

**Published:** 2012-10-29

**Authors:** Federica Riva, Eduardo Bonavita, Elisa Barbati, Marta Muzio, Alberto Mantovani, Cecilia Garlanda

**Affiliations:** ^1^Department of Veterinary Science and Public Health, University of MilanMilan, Italy; ^2^Department of Inflammation and Immunology, Humanitas Clinical and Research CenterRozzano, Italy; ^3^Department of Translational Medicine, University of MilanMilan, Italy; ^4^Division of Molecular Oncology, Ospedale San RaffaeleMilan, Italy

**Keywords:** cytokine, interleukin-1, toll like receptors, inflammation, infection, inflammation-associated cancer

## Abstract

Interleukin-1R like receptors (ILRs) and Toll Like Receptors (TLRs) are key receptors of innate immunity, inflammation, and orientation of the adaptive response. They belong to a superfamily characterized by the presence of a conserved intracellular domain, the Toll/IL-1R (TIR) domain, which is involved in the activation of a signaling cascade leading to activation of transcription factors associated to inflammation. The activation of inflammatory responses and immunity by ILRs or TLRs signaling is potentially detrimental for the host in acute and chronic conditions and is tightly regulated at different levels by receptor antagonists, decoy receptors or signaling molecules, and miRNAs. Recent evidence suggests that the ILRs family member TIR8 (also known as SIGIRR) is a regulatory protein acting intracellularly to inhibit ILRs and TLRs signaling. In particular, current evidence suggests that TIR8/SIGIRR dampens TLRs-mediated activation and inhibits signaling receptor complexes of IL-1 family members associated with Th1 (IL-18), Th2 (IL-33), and Th17 (IL-1) differentiation. Studies with Tir8/Sigirr-deficient mice showed that the ability to dampen signaling from ILRs and TLRs family members makes TIR8/SIGIRR a key regulator of inflammation. Here, we summarize our current understanding of the structure and function of TIR8/SIGIRR, focusing on its role in different pathological conditions, ranging from infectious and sterile inflammation, to autoimmunity and cancer-related inflammation.

## Introduction

**Figure 1:**
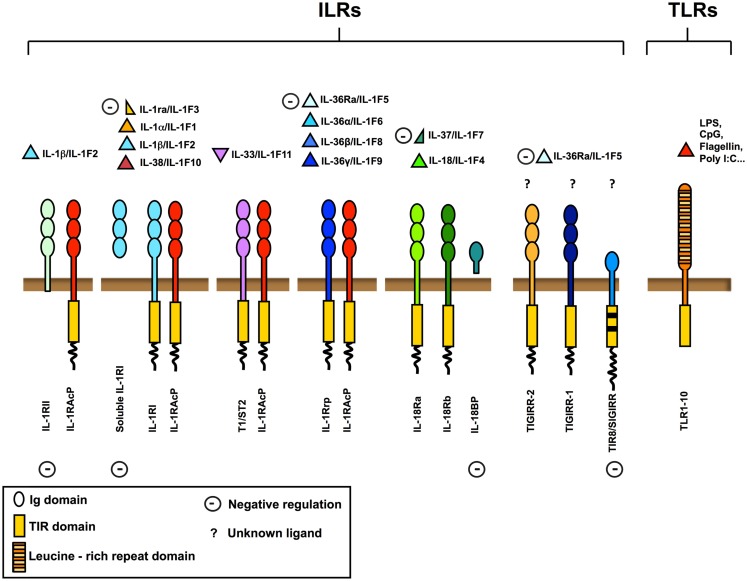
**The IL-1 receptor (ILRs) and Toll like receptor (TLRs) superfamily**. Ligands, receptors, adaptors, and extracellular and intracellular negative regulators are shown. The ILRs family includes the receptors (IL-1RI, IL-18R, T1/ST2, IL-1Rrp2) and the accessory proteins (IL-1RAcP) for IL-1, IL-18, IL-33, and other IL-1 family members (IL-36α, IL-36β, and IL-36γ). IL-1Ra, soluble IL-1RI, IL-36Ra, IL-37, IL-18 binding protein (IL-18BP), IL-1RII, and TIR8/SIGIRR are negative regulators acting at different levels, as receptor antagonists, decoy receptors, scavengers, or dominant negative. TIR8/SIGIRR, TIGIRR-1, and TIGIRR-2 are still orphan receptors. Microbial moieties (LPS, Poly I:C, CpG, Flagellin, and others) and necrotic cell-derived danger signals are the ligands for TLRs.

Interleukin-1R like receptors (ILRs) and Toll Like Receptors (TLRs) are key receptors of innate immunity and inflammation. They are members of a superfamily of phylogenetically conserved proteins characterized by the presence of a conserved intracellular domain, the Toll/IL-1R (TIR) domain (Figure 1).

Upon ligand binding, dimerization of receptor TIR domains, recruitment of TIR domain containing adapter proteins and activation of a signaling pathway occur. This pathway involves Myeloid differentiation factor 88 (MyD88), IL-1R associated kinases (IRAKs), and tumor necrosis factor receptor-associated factor 6 (TRAF6) and leads to activation of nuclear factor kappa B (NF-κB), activator protein-1 (AP-1), c-Jun N-terminal kinase (JNK), p38 mitogen-associated protein kinase, and members of the interferon regulatory factor family (O’Neill, 2006, 2008; Dinarello, 2009).

The family is subdivided in TLRs bearing leucine-rich repeats in the extracellular domain, and ILRs bearing Ig-like domains. Nowadays, 10 TLRs have been reported in humans and 12 in the mouse (Kang and Lee, 2011), whereas ILRs family includes 10 proteins. TLRs are receptors for specific pathogen associated molecular patterns (PAMPs) and of necrotic cell-derived danger signals (DAMPs) and act as sensors for microorganisms and tissue damage (Cavassani et al., 2008). The ILRs family includes components of signaling receptor complexes as well as molecules with regulatory function (IL-1RII and TIR8/SIGIRR). In particular TIR8, so called because at the time of cloning it was the eighth molecule with a TIR domain and also known as single immunoglobulin interleukin-1 receptor related molecule (SIGIRR), is a fringe member of the family with structural features incompatible with conventional signaling (Li and Qin, 2005; Garlanda et al., 2009; Figure 1).

Interleukin-1R like receptors ligand family includes pro-inflammatory molecules such as IL-1α and IL-1β (IL-1F1), IL-18/IL-1F4, IL-36α/IL-1F6, IL-36β/IL-1F8, and IL-36γ/IL-1F9 (Dinarello et al., 2010; Boraschi et al., 2011). Other members of IL-1 family show anti-inflammatory activity. IL-1 receptor antagonist (IL-1Ra)/IL-1F3 binds to IL-1RI inhibiting the recruitment of IL-1RAcP and compete with IL-1α and IL-1β for receptor binding (Dinarello, 2000); IL-36Ra binds IL-1Rrp2 antagonizing IL-36α, IL-36β, and IL-36γ; IL-37 produces anti-inflammatory effects; and finally IL-33 binds to T1/ST2, recruits IL-1RAcP and induces the expression of anti-inflammatory cytokines (Schmitz et al., 2005).

Uncontrolled or deregulated activation of ILRs- or TLRs-dependent inflammatory and immune responses can be detrimental for the host and potentially cause tissue damage and acute or chronic inflammatory disorders. The activation of inflammatory responses and immunity by ILRs or TLRs signaling is tightly regulated at different levels. For instance, IL-1Ra and IL-36Ra are polypeptide antagonists for IL-1RI and IL-1Rrp2, respectively (Towne et al., 2004; Costelloe et al., 2008; Aksentijevich et al., 2009; Dinarello, 2009; Reddy et al., 2009). Decoy receptors, such as IL-1RII, bind ligands that are no longer available for the transducing receptors or form dominant negative non-signaling complexes with AcPs (Mantovani et al., 2001). IRAK-M and MyD88s are negative regulators of ILRs and TLRs signaling, acting intracellularly (Kobayashi et al., 2002; Janssens et al., 2003). TIR8/SIGIRR, which will be discussed in detail below, inhibits the activation of the signaling pathway by TLRs and IL-1R by interfering with the association of adaptor molecules to the receptor complex. Finally, a novel level of feedback system has been identified, consisting of targeting of ILRs or TLRs signaling proteins or transcription factors by miRNAs. In particular, miR-155, miR-21, miR-146a, miR-132, miR-9, and miR-147 have all been significantly implicated in the immune response initiated by IL-1R or TLRs (Bazzoni et al., 2009; Nahid et al., 2011; Quinn and O’Neill, 2011). For instance, the induction of transcription of miR-146a or miR-9 by LPS, tumor necrosis factor alpha (TNFα) and IL-1α is dependent on NF-κB, and in turn, miR-146a potentially targets TRAF6 and IRAK-1, whereas miR-9 targets the NFkB1 transcript, dampening the immune response (Bazzoni et al., 2009; Quinn and O’Neill, 2011).

Here, we summarize our current understanding of the structure and function of TIR8/SIGIRR, focusing on its regulatory role in different pathological disorders dependent on ILRs and TLRs activity, ranging from inflammation in infectious and sterile conditions, to autoimmunity and cancer-related inflammation.

## TIR8/SIGIRR Gene and Protein

TIR8/SIGIRRis an orphan receptor. The human gene is localized on chromosome 11, band p15.5, and encompasses 10 exons spanning about 11700 bp, whereas the murine gene is localized on chromosome 7, band F4, and includes nine exons spanning about 4000 bp (Thomassen et al., 1999). The human protein is 410 amino acid-long and is organized in an extracellular domain comprising of a single Ig domain, in contrast with others ILRs family members which have three, a transmembrane domain, an intracellular conserved TIR domain, and a peculiar terminal 95 aa long tail, which is not present in other mammal TIR family members. Two residues of the TIR domain (Ser447 and Tyr536) considered necessary for signal transduction of IL-1R1 are replaced by Cys222 and Leu305 in TIR8/SIGIRR, but the functional relevance of this replacement has not been addressed. TIR8/SIGIRR is extensively *N*- and *O*-glycosylated. Indeed five potential *N*-glycosylated sites in human and four in mouse are present in the extracellular portion of the molecule (Thomassen et al., 1999; Lech et al., 2007).

*TIR8/SIGIRR* shows a conserved sequence and pattern of expression among vertebrates, from chicken to humans (Riva et al., 2009). In particular, protein sequences of human and mouse TIR8/SIGIRR are 82% identical.

Little is known about *TIR8/SIGIRR* phylogenetic evolution, but the recent discovery in teleost fish of DIGIRR is helpful in defining the evolutionary history of TIR family members. DIGIRR presents two Ig-like domains in its extracellular region, a Arg-Tyr-mutated TIR domain and a unique expression within the Golgi apparatus. *In vitro* experiments demonstrate that DIGIRR is a negative regulator of LPS and IL-1β-mediated NF-κB activation (Gu et al., 2011). The authors suggest that DIGIRR could represent a “transitional molecule” between a potent receptor such as IL-1R1 and a negative regulator such as TIR8/SIGIRR.

TIR8/SIGIRR is ubiquitously expresses in tissues, in particular in kidney, digestive tract, liver, lung, and lymphoid organs (Thomassen et al., 1999; Polentarutti et al., 2003). Particularly, in kidney it is expressed on the luminal border and basolateral membrane of proximal tubular cells (Polentarutti et al., 2003; Lech et al., 2007), in the intestinal tract by epithelial cells and in lymphatic organs by NK cells, B lymphocytes, monocytes, and immature dendritic cells (DCs)(Polentarutti et al., 2003; Garlanda et al., 2004; Lech et al., 2007; Xiao et al., 2007), in the lung by bronchial epithelium, blood endothelial cells, and leukocytes (Veliz Rodriguez et al., 2012).

Mechanisms of regulation of TIR8/SIGIRR expression are still poorly defined (Table 1).

**Table 1:** **Regulation of TIR8/SIGIRR expression**.

Stimuli	Organism	Cell type	mRNA/protein	Reference
**UPREGULATION**				
Th2-polarization	Mouse	T lymphocytes	Protein	Bulek et al. (2009)
Vasoactive intestinal peptide	Mouse	Macrophages and langerhans cells	mRNA	Jiang et al. (2012)
LPS	Mouse	Payer’s patch DCs	mRNA	Davies et al. (2010)
**DOWNREGULATION**				
LPS	Mouse and human	Epithelial cells, DC, monocytes, polymorphonuclear granulocytes	mRNA, protein	Polentarutti et al. (2003), Wald et al. (2003), Huang et al. (2006), Garlanda et al. (2004), Kadota et al. (2010)
Flagellin, bacterial infection	Mouse and human	Intestinal epithelial cells	Protein	Khan et al. (2010)
Psoriatic arthritis	Human	Peripheral blood leukocytes	mRNA	Batliwalla et al. (2005)
Necrotizing enterocolitis	Human	Fetal enterocytes	mRNA	Nanthakumar et al. (2011)
Asymptomatic bacteriuria	Human	Neutrophil polymorphonuclear granulocytes	Protein	Ragnarsdottir et al. (2007)
*P. aeruginosa* acute lung infection	Mouse	Respiratory epithelium, polymorphonuclear granulocytes	mRNA	Veliz Rodriguez et al. (2012)
*Toxoplasma gondii*	Mouse	Intestinal epithelial cells	mRNA	Gopal et al. (2008)

Kodota et al. recently showed that *TIR8/SIGIRR* proximal promoter presents a binding site for the transcription factor SP1, a zinc finger proteins, which binds directly to DNA and enhances *TIR8/SIGIRR* gene transcription. In the presence of LPS, SP1 binding to *TIR8/SIGIRR* promoter consensus sites was reduced, and consequently *TIR8/SIGIRR* expression was transiently inhibited in epithelial cells (Kadota et al., 2010). These data potentially explain previous findings showing that human and murine *TIR8/SIGIRR* expression was usually found down-regulated by LPS stimulation or in other inflammatory conditions compared to homeostatic conditions (Polentarutti et al., 2003; Wald et al., 2003; Huang et al., 2006). For instance, ulcerative colitis in human and colitis in mouse were associated with reduced TIR8/SIGIRR expression by epithelial cells (Kadota et al., 2010). Bacterial infection of intestinal epithelial cells and exposure to flagellin transiently decreased TIR8/SIGIRR protein expression. Conversely, stable overexpression of TIR8/SIGIRR diminished NF-κB–mediated IL-8 responses to TLRs ligands (Khan et al., 2010). In psoriatic patients peripheral blood cells expressed decreased levels of *TIR8/SIGIRR* and other anti-inflammatory molecules (Batliwalla et al., 2005). Nanthakumar et al. (2011) demonstrated that *TIR8/SIGIRR*, together with other anti-inflammatory genes, was expressed at low levels in fetal human enterocytes and at lower levels in necrotizing enterocolitis intestinal cells, whereas pro-inflammatory genes were present at high levels, potentially explaining the excessive inflammatory response of the immature intestine. In asymptomatic bacteriuria patients, *TIR8/SIGIRR* and *TLR4* expression were significantly lower than in the age-matched control subjects (Ragnarsdottir et al., 2007). In mouse, *Tir8/Sigirr* mRNA was down-regulated upon acute lung infection by *Pseudomonas aeruginosa* in the lung and in neutrophils (Veliz Rodriguez et al., 2012) or in intestinal epithelial cells upon *Toxoplasma gondii* (Gopal et al., 2008). However, Adib-Conquy et al. (2006) reported that human monocytes up-regulated the *TIR8/SIGIRR* transcript during sepsis and sterile systemic inflammation, which was associated to reduced TNFα and enhanced IL-10 production in response to LPS and Pam3CysSK4.

Stimuli inducing *TIR8/SIGIRR* expression are not well defined yet and mechanisms involved are not known. In T lymphocytes, Th2-polarization induced higher levels of *TIR8/SIGIRR* expression than Th1-polarization or non-differentiating conditions (Bulek et al., 2009). The neuropeptide vasoactive intestinal peptide (VIP) was found to up-regulate *Tir8/Sigirr* in a cAMP-independent manner in the cornea of *P. aeruginosa* infected mice as well as in macrophages and Langerhans cells (Jiang et al., 2012). Recently, the probiotic microorganism *Lactobacillus jensenii* was found to up-regulate *TIR8/SIGIRR*, *A20*, and *IRAK-M* in porcine Payer’s patch antigen presenting cells through TLR2, and to increase the expression of IL-10 and TGF-β thus inducing tolerogenic properties (Villena et al., 2012).

Finally, *in vitro* and *in vivo* experiments showed that LPS-induced up-regulation of *Tir8/Sigirr*, *tollip*, and *T1/ST2* messenger RNA in murine Payer’s patch DCs, but not in spleen DCs, suggesting that Payer’s patch DCs use these molecules in their arsenal to prevent the initiation of TLRs signaling (Davies et al., 2010).

## Regulation of ILRs and TLRs Signaling by TIR8/SIGIRR

**Figure 2:**
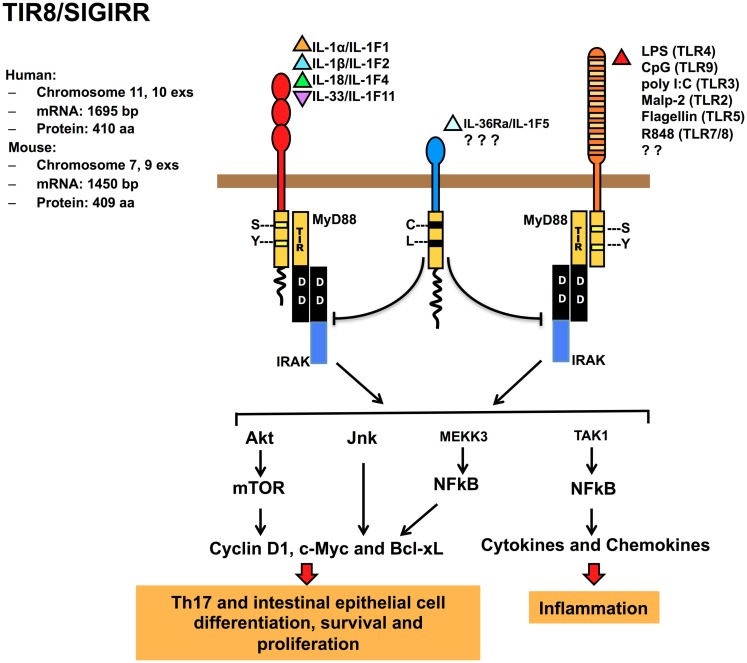
**Regulatory function of TIR8/SIGIRR in signaling**. TIR8/SIGIRR is a receptor composed by a single extracellular Ig domain (amino acids 17–112), a transmembrane domain (amino acids 117–139); an intracellular conserved TIR domain (amino acids 166–305), and a 95 amino acid-long intracellular tail. Two conserved amino acid (Ser, Tyr in TIR domain) necessary for IL-1R-signaling are replaced in TIR8/SIGIRR (Cys 222, Leu 305) potentially leading to a non-conventional activation. TIR8/SIGIRR is an orphan receptor, but IL-36Ra has been proposed as a TIR8/SIGIRR ligand in glial cells. TIR8/SIGIRR inhibits ILR (IL-1RI, IL-18R, T1/ST2) and TLR (TLR2, TLR3, TLR4, TLR5, TLR7/8, TLR9, and possibly others) signaling and NF-κB activation. In T cells and epithelial cells, TIR8/SIGIRR inhibits IL-1-dependent activation of the Akt-mTOR pathway and of JNK.

TIR8/SIGIRR lacks a specific ligand, even if it was demonstrated that glial cell TIR8/SIGIRR interacts with IL-36Ra regulating inflammatory response in the brain (Costelloe et al., 2008). TIR8/SIGIRR inhibits NF-κB and JNK activation dependent on ILRs or TLRs family member activation, but not TNFα-dependent NF-κB activation or IFNγ-dependent STAT1 activation (Wald et al., 2003; Garlanda et al., 2004). The inhibitory activity is exerted on IL-1RI, IL-18R, T1/ST2, TLR4, TLR7, and TLR9 (Wald et al., 2003; Garlanda et al., 2004; Qin et al., 2005; Lech et al., 2007, 2008; Bulek et al., 2009). TIR8/SIGIRR regulates also TLR3 and TLR1/2-dependent signaling in kidney monocytes (Lech et al., 2007; Figure 2).

Little is known about the interaction between TIR8/SIGIRR and other members of the superfamily and the data are sometimes contradictory. This could be explained by specific expression of TIR8/SIGIRR or other receptor complex components in different cell types or by posttranscriptional modifications, such as glycosylation, that modify biological function of TIR8/SIGIRR (Garlanda et al., 2004).

It has been proposed that the extracellular Ig-like domain of TIR8/SIGIRR interferes with the dimerization of IL-1RI and IL-1RAcP. In addition, the TIR8/SIGIRR cytoplasmic TIR domain sequesters proximal TIR-containing adaptor molecules, such as IRAK and TRAF6 after IL-1 stimulation. Mutagenesis studies showed that only the TIR domain (excluding the C-tail part) was necessary for TIR8/SIGIRR to inhibit TLR4 signaling (Wald et al., 2003; Qin et al., 2005). In contrast, T1/ST2 interacts with both TIR8/SIGIRR extracellular and intracellular domains, even if the TIR domain is crucial for TIR8/SIGIRR activity (Bulek et al., 2009).

Gong et al. (2010) addressed by a computational approach the molecular mechanism for the interaction of TIR8/SIGIRR and TLR4 or TLR7 and constructed a three-dimensional model for the TLR4, TLR7, MyD88, and TIR8/SIGIRR TIR domains. Receptor activation would trigger the formation of TLR4 and TLR7 TIR dimers recruiting MyD88 TIR dimers resulting in a signaling tetramer, in line with previous data (Loiarro et al., 2005; Nunez Miguel et al., 2007). The model proposed revealed that TIR8/SIGIRR binds through its BB-loop region to TLR4 and TLR7 by occupying their self-interacting sites and that the proper shape and electric environment of the MyD88-binding pocket are completely disturbed. In addition, TIR8/SIGIRR replaces a MyD88 monomer, interrupting the MyD88 homodimer formation (Gong et al., 2010). The BB-loop region is highly conserved in TIR-containing molecules and plays a crucial role in dimer formation (Gay et al., 2011). Thus, the interference exerted by TIR8/SIGIRRBB-loop region prevents the dimerization of the components of the receptor complex (receptor, accessory protein and adapter molecule), abolishing the signal transduction. In contrast, TIR8/SIGIRR unique C-tail is distant from the active BB-loop, consistent with the observation that this tail is not required for TIR8/SIGIRR inhibitory effect on TLRs signaling (Qin et al., 2005).

TIR8/SIGIRR also modulates IL-1-induced phosphorylation of JNK and mTOR kinase in Th17 lymphocytes, playing a non-redundant role in controlling mTOR-dependent Th17 differentiation, proliferation, and cytokine production (Gulen et al., 2010). On the same line, TIR8/SIGIRR was found to regulate IL-1- or commensal-TLRs-dependent activation of the Akt-mTOR axis in intestinal epithelial cells. The Akt-mTOR axis promotes cycle progression through its impact on posttranscriptional control of key cell cycle regulators and consequent genetic instability (Xiao et al., 2010; Figure 2). These findings open a new scenario for TIR8/SIGIRR acting as a modulator of autoimmune diseases and as tumorigenesis suppressor (see below).

## Role of TIR8/SIGIRR in Infection-Dependent Inflammation

**Figure 3:**
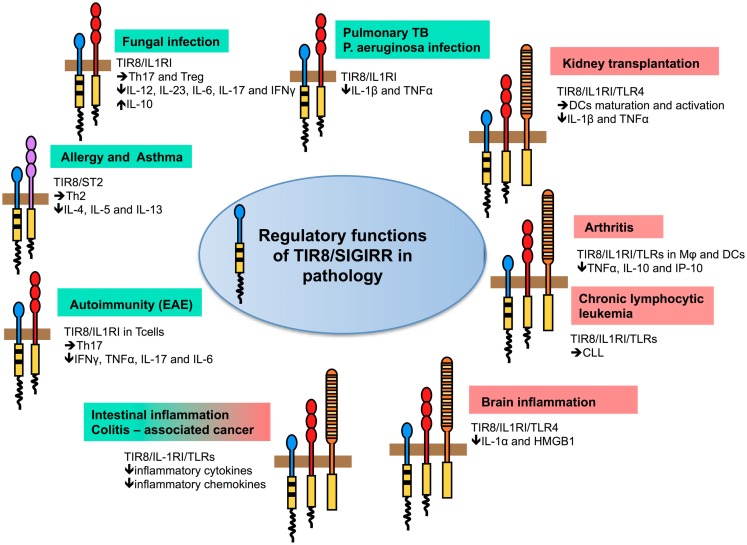
**Regulatory function of TIR8/SIGIRR in pathology**. TIR8/SIGIRR inhibits ILR (IL-1RI, IL-18R, T1/ST2) and TLR (TLR4, TLR7, TLR9, and possibly others) signaling and NF-κB activation. Gene-targeted mice demonstrate that Tir8/Sigirr acts as a non-redundant negative regulator *in vivo* in different inflammatory conditions dependent on ILR and TLR activation. For some of these, the pathway prominently regulated by TIR8/SIGIRR and playing a relevant role in the pathogenesis of the disease has been defined. For instance, in autoimmunity (EAE) TIR8/SIGIRR regulates IL-1-dependent Th17 cell differentiation, survival, and proliferation; in asthma TIR8/SIGIRR regulates the IL-33/ST2 pathway and Th2 responses; in models of infections (*Candida albicans*, *Aspergillus fumigatus*, *Mycobacterium tuberculosis*, and *Pseudomonas aeruginosa*) TIR8/SIGIRR regulates IL-1RI signaling controlling Th17 and Treg responses (fungal infections) and pro-inflammatory cytokine production (bacterial infections). In colitis-associated colon cancer, TIR8/SIGIRR plays a major role in controlling microbial flora-dependent activation of TLRs, but the control on ILRs-dependent inflammation can not be excluded. In other contexts (chronic lymphocytic leukemia, arthritis, kidney transplantation, and brain inflammation), the molecular pathway regulated by TIR8/SIGIRR remains to be addressed.

In different infectious conditions, TIR8/SIGIRR emerged as a non-redundant molecule involved in dampening inflammation and tissue damage by controlling TLRs-, but in particular ILRs-induced inflammatory response to pathogens (Figure 3; Table 2).

**Table 2:** **Regulatory functions of TIR8/SIGIRR in disease**.

	Reference
**INFECTION-DEPENDENT INFLAMMATION**	
*Mycobacterium tuberculosis* lung infection	Garlanda et al. (2007a)
Mucosal and disseminated *Candida albicans* infections	Bozza et al. (2008)
*Aspergillus fumigatus* lung infections	Bozza et al. (2008)
*Pseudomonas aeruginosa* keratitis	Huang et al. (2006)
*Pseudomonas aeruginosa* lung infection	Veliz Rodriguez et al. (2012)
Dextran sodium sulfate (DSS) colitis model	Garlanda et al. (2004), Xiao et al. (2007)
Survival to endotoxin	Wald et al. (2003)
LPS-dependent acute lung injury	Chen et al. (2011)
**AUTOIMMUNE DISEASES AND ALLERGY**	
Experimental autoimmune encephalomyelitis (EAE)	Gulen et al. (2010)
Systemic lupus erythematosus(lpr and pristane models)	Lech et al. (2008, 2010)
Rheumatoid arthritis	Drexler et al. (2010)
OVA-induced allergic pulmonary inflammation	Bulek et al. (2009)
**KIDNEY STERILE INFLAMMATION**	
Postischemic acute renal failure	Lech et al. (2009)
Kidney transplantation	Noris et al. (2009)
**BRAIN INFLAMMATION**	
LPS-dependent sickness behavior and age-related neuroinflammation	Watson et al. (2010)
Cognitive and synaptic functions	Costelloe et al. (2008), Costello et al. (2011)
**CANCER**	
Colitis-associated cancer (AOM/DSS model)	Garlanda et al. (2007b), Xiao et al. (2007)
Spontaneous intestinal cancer (Apc^min/+^ model)	Xiao et al. (2010)
Chronic lymphocytic leukemia (TCL1 transgenic mouse model)	Bertilaccio et al. (2011)

In *Mycobacterium tuberculosis* lung infection, Tir8/Sigirr-deficient mice showed exaggerated susceptibility to mortality, associated to overwhelming systemic inflammatory response, despite there was no difference in tissue bacterial load compared to control mice (Garlanda et al., 2007a). Indeed, treatment of Tir8/Sigirr-deficient mice with blocking anti-IL-1 and anti-TNFα antibodies during *M. tuberculosis* infection significantly prolonged survival (Garlanda et al., 2007a).

In *Candida albicans* or *Aspergillus fumigatus* infections, Tir8/Sigirr-deficient mice showed increased susceptibility to mucosal and disseminated or lung infections, respectively, in terms of mortality and fungal burden, which were associated to increased IL-1 signaling and heightened Th17 cell response (Bozza et al., 2008). The IL-17 pathway plays an important role in the protective antifungal host defense (Van De Veerdonk et al., 2011), however, rigorous control of the inflammatory Th17 response is necessary to prevent immunopathology (Park et al., 2005; Zelante et al., 2007). Thus, Tir8/Sigirr-deficient mice phenotype was possibly dependent on IL-1-mediated deregulated Th17 responses.

In a model of keratitis induced by *P. aeruginosa*, TIR8/SIGIRR was involved in down-regulating Th1 immunity and associated IL-1RI and TLR4-activation, thus preventing tissue damage and promoting resistance to infection (Huang et al., 2006). On the same line, in acute lung infections caused by *P. aeruginosa*, a Gram-negative pathogen responsible of life-threatening infections in immunocompromised individuals and cystic fibrosis patients, Tir8/Sigirr-deficiency was associated to increased susceptibility in terms of mortality and bacterial load, and to exacerbated local and systemic production of pro-inflammatory cytokines and chemokines. IL-1RI-deficiency rescued the phenotype observed in Tir8/Sigirr-deficient mice. This suggests the non-redundant role of TIR8/SIGIRR in negatively regulating IL-1RI signaling, which plays a major role in the pathogenesis of *P. aeruginosa* infection (Veliz Rodriguez et al., 2012).

Thus, in both tuberculosis, *Candida*, and *P. aeruginosa* infections, the major protective role identified for TIR8/SIGIRR was the suppression of excessive IL-1-dependent inflammatory pathology, since inhibition of IL-1-signaling was sufficient to rescue the phenotype of Tir8/Sigirr-deficient mice in these infections.

In dextran sodium sulfate (DSS) colitis model, Tir8/Sigirr-deficient mice exhibited a dramatic intestinal inflammation compared to wild type mice in terms of weight loss, intestinal bleeding, local tissue damage, and mortality, which was associated to increased leukocyte infiltration and pro-inflammatory cytokine, chemokine, and prostaglandin production (Garlanda et al., 2004; Xiao et al., 2007). In homeostatic conditions, TIR8/SIGIRR was described to be involved in controlling gut homeostasis. Indeed, TLRs signaling activated by commensal gut microflora provides the survival signal for intestinal epithelial cells (Rakoff-Nahoum et al., 2004; Karin et al., 2006), and Tir8/Sigirr-deficiency was associated to increased proliferation in colon crypts and decreased apoptosis (Xiao et al., 2007). Tir8/Sigirr-deficient colon epithelial cells revealed constitutive NF-κB and JNK activation and up-regulated expression of Cyclin D1 and Bcl-xL, which were further increased by treatment with IL-1 or LPS and returned to the control level after depletion of the commensal bacteria (Xiao et al., 2007). This spontaneous phenotype was not confirmed in other studies (Garlanda et al., 2004, 2007b), possibly a reflection of animal house-dependent variation in microbiota.

Finally, after endotoxin challenge, survival was reduced in Tir8/Sigirr-deficient mice on a BALB/c background (Wald et al., 2003), and overexpression of Tir8/Sigirr in lung epithelial cells suppressed the inflammatory reaction and improved the survival of BALB/c mice in a model of LPS-dependent Acute Lung Injury (Chen et al., 2011). However, Tir8/Sigirr-deficient mice on a C57BL/6 × 129/Sv background showed normal systemic or local inflammatory reactions to LPS (Garlanda et al., 2004).

The relevance in human of data obtained in mice is supported by a recent genetic analysis suggesting for the first time associations of TIR8/SIGIRR gene region SNPs with susceptibility to an infectious disease, in particular with the development of both pulmonary tuberculosis and tuberculous meningitis in independent cohorts of Vietnamese subjects (Horne et al., 2012).

## Role of TIR8/SIGIRR in Autoimmune Diseases and Allergy

Members of the ILRs family have emerged as key players in T cell polarization (Schmitz et al., 2005; Acosta-Rodriguez et al., 2007). In particular, IL-1 has been identified as a critical factor in the differentiation and activation of Th17 cells, which mediate the development of autoimmune and inflammatory diseases, such as rheumatoid arthritis (RA), systemic lupus erythematosus, multiple sclerosis, psoriasis, and inflammatory bowel disease (IBD; Mills, 2011); whereas, IL-33 has been implicated in the initiation and propagation of Th2 immune responses, involved in allergy and asthma (Lloyd, 2010).

Gulen et al. (2010) recently showed that TIR8/SIGIRR was induced during Th17 cell differentiation and that it suppressed Th17 cell differentiation, proliferation, and cytokine production. TIR8/SIGIRR was shown to act through direct inhibition of multiple IL-1-dependent signaling pathways in T cells, in particular mTOR, a key transcription factor in IL-1-dependent Th17 cell proliferation. TIR8/SIGIRR suppressed IL-1 signaling during initial differentiation of Th17 cells as well as in differentiated effector Th17 cells, acting as key regulator to prevent overactivation of Th17 cell-mediated pathogenic effects. Indeed, regulation of IL-1R-mTOR pathway of Th17 development and activation by TIR8/SIGIRR was critical for the control of Th17 cell-dependent development of central nervous system (CNS) autoimmune inflammation (Figure 3; Table 2). In the absence of this IL-1 regulatory mechanism, Tir8/Sigirr-deficient mice were more susceptible to experimental autoimmune encephalomyelitis (EAE) resulting from hyperactivation of Th17 cells upon immunization with myelin oligodendrocyte glycoprotein (MOG) peptide, which infiltrated the CNS in greater numbers and showed enhanced pathogenic functions compared to wild type Th17 cells (Gulen et al., 2010).

In addition to IL-1 signaling in Th17 cells, TLRs signaling in innate immune cells, antigen presenting cells, and T cells plays a pathogenetic mechanism in different autoimmune diseases (Mills, 2011). In particular, immune complexes containing the lupus autoantigen U1snRNP or nucleosomes activate DCs and autoreactive B-cells via TLR7 and TLR9, respectively, contributing in this way to pathogenesis of systemic lupus erythematosus (Leadbetter et al., 2002; Marshak-Rothstein and Rifkin, 2007). Genetic approaches showed that Tir8/Sigirr-deficiency alone did not induce autoimmunity against DNA, however, the deficiency of Tir8/Sigirr in C57BL/6lpr/lpr mice, which develop delayed autoimmunity due to impaired Fas-induced apoptosis of autoreactive B and T cells, caused massive lymphoproliferation, peribronchial inflammation and mesangioproliferative glomerulonephritis (Lech et al., 2008). This autoimmune tissue damage was associated with increased production of autoantibodies (anti-dsDNAIgG, anti-nucleosome, anti-Sm antigen, anti-snRNP, and rheumatoid factor) and early development of hypergammaglobulinemia. Tir8-deficient lpr/lpr mice showed enhanced activation of DCs to complexed lupus autoantigens, increased production of pro-inflammatory cytokines (e.g., CCL2, IL-6, and IL-12p40) and B-cell survival factors (e.g., Baff/BlyS and Bcl-2), increased B cell proliferation upon exposure to RNA and DNA immune complexes and other TLRs agonists, and production of lupus autoantibodies. Thus, the pathogenetic mechanisms underlying the susceptibility of Tir8/Sigirr-deficient mice in lupus include increased activation of antigen presenting cells that handle autoantigens, proliferation of autoreactive B lymphocytes, and production of immunoregulatory factors. On the same line, Tir8/Sigirr-deficiency caused increased susceptibility to lupus nephritis in a model, which mimics environmentally induced autoimmunity: intraperitoneal injection of hydrocarbon oil (pristane) causes persistent abundance of apoptotic peritoneal cells, chronic granulomatous peritonitis, ectopic lymphoid tissue formation, and evolution of antinuclear antibodies, immune complex disease, and lupus nephritis. In this model, a major pathogenetic role is played by TLR7 signaling and type I interferon (Savarese et al., 2008). Tir8/Sigirr protected from hydrocarbon oil-induced lupus by suppressing the TLR7-mediated activation of DCs and expansion of IgG and RNA autoreactive lymphocyte clones (Lech et al., 2010). Structure model prediction identified the BB-loop of TIR8/SIGIRR intracellular TIR domain to interact with TLR7 and MyD88 (Gong et al., 2010) and indeed, BB-loop deletion was sufficient to completely abrogate TIR8/SIGIRR inhibitory effect on TLR7 signaling in DCs (Lech et al., 2010). The relevance of genetic variants of the TIR8/SIGIRR gene in susceptibility to SLE was recently investigated in a large European-descent population, but the analysis was restricted to a single missense polymorphism (rs3210908), which results in the replacement of a glutamine (Gln) for arginine (Arg) at amino acid 312 (Q312R) and it did not reveal any association between *TIR8/SIGIRR* gene variants and SLE (Sanchez et al., 2012).

Increasing evidence implicate TLRs signaling also in RA (Sacre et al., 2007; Abdollahi-Roodsaz et al., 2009; Drexler and Foxwell, 2010). Overexpression of TIR8/SIGIRR in RA synovial cells led to a significant inhibition of spontaneous release of pro-inflammatory mediators, suggesting that either ILRs and/or TLRs signaling was at least partly responsible for the chronic production of those mediators in RA (Drexler et al., 2010). The role of TIR8/SIGIRR as an inhibitor of inflammation was confirmed *in vivo*, since Tir8/Sigirr-deficient mice developed a more severe disease in both the zymosan-induced arthritis and collagen antibody-induced arthritis models, which was due to increased cellular infiltration into the affected joints (Drexler et al., 2010). IL-1 plays a significant role in zymosan-induced arthritis model, and IL-1Ra reduced the disease severity in Tir8/Sigirr-deficient mice, but did not completely rescued the phenotype, suggesting that additional factors, including TLRs ligands, are driving pathology and are under Tir8/Sigirr control (Figure 3; Table 2). In agreement with this study, a gene expression study showed that *TIR8/SIGIRR* was one among the genes with the most significantly reduced expression in peripheral blood cells of patients with psoriatic arthritis compared to control subjects. *TIR8/SIGIRR* clustered with other genes involved in downregulation or suppression of innate and acquired immune responses, suggesting inappropriate control that favors pro-inflammatory responses (Batliwalla et al., 2005).

T1/ST2, the receptor of IL-33 preferentially expressed in Th2 cells, is a further ILRs controlled by TIR8/SIGIRR. IL-33 plays a major role in Th2 responses by inducing Th2 cytokines IL-4, IL-5, IL-13, splenomegaly, eosinophilia, and allergy (Schmitz et al., 2005). TIR8/SIGIRR was shown tobe expressed during Th2-polarization and to inhibit IL-33-and T1/ST2-mediated signaling and Th2 cytokine (IL-4, IL-5, and IL-13) production *in vitro* and *in vivo* (Bulek et al., 2009). Indeed, Tir8/Sigirr-deficient mice showed hyper responsiveness to IL-33 with increased serum levels of IL-5 and IL-13, splenomegaly, lung inflammation, and exacerbated Th2 responses in OVA-induced allergic pulmonary inflammation. These results indicate that Tir8/Sigirr serves as a negative feedback control in Th2-polarization and restimulation, thus controlling allergic inflammatory responses (Bulek et al., 2009; Figure 3; Table 2). However, a genetic analysis performed on about 850 asthma patients and 640 healthy subjects failed to identify any association between 12 *TIR8/SIGIRR* polymorphisms or haplotypes identified in this Japanese population with asthma susceptibility or asthma-related phenotype (Nakashima et al., 2006).

## Role of TIR8/SIGIRR in Kidney Sterile Inflammation

Among solid organs and tissues, TIR8/SIGIRR is highly expressed in the kidney, in particular by tubular epithelial cells, DCs, and macrophages, but functional activity of TIR8/SIGIRR in suppressing TLRs-induced expression of pro-inflammatory cytokines has been demonstrated in renal immune cells but not in renal parenchymal cells (Lech et al., 2007). The relevance in pathology of TIR8/SIGIRR expression in kidney has been demonstrated in different conditions, such as lupus nephritis (Lech et al., 2008, 2010; discussed above) and postischemic acute renal failure or kidney transplantation (Lech et al., 2009; Noris et al., 2009), conditions associated to TLRs activation by nucleosomes and DAMPs released during ischemic cell necrosis, respectively (Figure 3; Table 2).

Postischemic acute renal failure represents a state of sterile inflammation, in which DAMPs activate innate immune elements, mostly neutrophils and macrophages, which rather enhances the subsequent tissue injury than promoting the healing phase, in particular through TLR4 and TLR2 (Leemans et al., 2005). In a postischemic renal failure model, intrarenal DCs or macrophages were excessively activated in Tir8/Sigirr-deficient mice (Lech et al., 2009). Hyper activation of myeloid cells increased intrarenal cytokine and chemokine production and consequently, leukocyte recruitment, and renal injury. Renal ischemia/reperfusion studies with chimeric mice confirmed the primary role of Tir8/Sigirr in suppression of hematopoietic cell activation as compared to tubular epithelial cell, since lack of Tir8/Sigirr in hematopoietic cells largely reproduced the phenotype of renal IR injury seen in Tir8/Sigirr-deficient mice.

In the same line, Noris et al. (2009) showed that the early post transplant kidney inflammatory response was more severe in Tir8/Sigirr-deficient grafts, as shown by increased numbers of infiltrating neutrophils and macrophages and higher TNFα and chemokine expression, and was followed by an amplified adaptive immune response against donor antigens, so that acute allograft rejection occurred within 7–10 days after transplantation. Indeed, the lack of Tir8/Sigirr in the kidney graft led to amplification of all downstream effects of ischemia/reperfusion-induced TLRs/IL-1R-dependent inflammation in the graft. The expansion and maturation of DCs in the graft, Th1 T cell priming, and block of Treg development finally resulted in acute rejection, thus demonstrating a role for renal Tir8/Sigirr as a first-line regulation of allogeneic immune response *in situ* (Noris et al., 2009).

In contrast, lack of Tir8/Sigirr did not affect the intrarenal mRNA expression of pro-inflammatory chemokines, profibrotic mediators, or markers of renal fibrosis after unilateral ureter obstruction, nor the number of intrarenal macrophages and myofibroblasts or tissue remodeling in post obstructive kidneys (Skuginna et al., 2011). These results are in agreement with data showing that TLR2-, TLR9-, and MyD88-signaling do not significantly contribute to this model (Chowdhury et al., 2010).

## Role of TIR8/SIGIRR in Brain Inflammation

TIR8/SIGIRR is also present in brain (Polentarutti et al., 2003; Costelloe et al., 2008) where it is expressed on neurons, microglia, and astrocytes (Andre et al., 2005).

TIR8 was shown to play a functional role in suppressing microglial activation by LPS and overexpression of CD40 and ICAM-1 or the production of TNFα, IL-6, and other pro-inflammatory mediators, both *in vitro* and *in vivo*, in hippocampal tissue. The effect of LPS on exploratory behavior, the so called sickness behavior, as well as age-related neuroinflammation were also accentuated in Tir8/Sigirr-deficient mice and were associated with increased hippocampal expression of CD14 and TLR4, and NF-κB activation (Watson et al., 2010). In addition, in the absence of any external inflammatory stimulus, cognitive, and synaptic functions, such as novel object recognition, spatial reference memory, and long-term potentiation (LTP), were defective in Tir8/Sigirr-deficient mice and were associated with up-regulation of IL-1RI- and TLR4-mediated signal transduction in hippocampus (Costello et al., 2011; Table 2). IL-1ra, an anti-TLR4 antibody, and also the inhibition of JNK and NF-κB restored the decrease in synaptic functions in Tir8/Sigirr-deficient mice, demonstrating the key role of IL-1RI-and TLR4-activation by IL-1α and high mobility group box 1 (HMGB1), respectively, in this model. These findings highlighted the functional role of Tir8/Sigirr in regulating inflammatory mediated synaptic and cognitive decline, and described evidence of the key role of HMGB1 in this process (Costello et al., 2011).

Finally, it has been suggested that IL-36Ra has anti-inflammatory effects in the brain since it abrogated IL-1 and LPS-induced inflammatory responses specifically in glial cells and IL-1- or LPS-induced inhibition of LTP and the associated increase in IL-1β concentration. These effects of IL-36Ra depended on IL-4 production and were absent in mixed glia prepared from Tir8/Sigirr-deficient mice, suggesting that the anti-inflammatory effects of this cytokine are mediated, at least in part, by TIR8/SIGIRR (Costelloe et al., 2008).

## Role of TIR8/SIGIRR in Intestinal Cancer

The connection between cancer and inflammation is well recognized in tumors epidemiologically linked to inflammatory processes. In addition, an inflammatory component is present in the microenvironment of most neoplastic tissues and includes the infiltration of white blood cells, prominently tumor-associated macrophages (TAM); the presence of inflammatory cytokines (e.g., TNFα, IL-1, IL-6, IL-23, IL-17, chemokines, such as CCL2); the occurrence of tissue remodeling and angiogenesis (Mantovani et al., 2008; Colotta et al., 2009; Biswas and Mantovani, 2010; Ben-Neriah and Karin, 2011). Two pathways link inflammation and cancer: in the intrinsic pathway, activation of different classes of oncogenes drives the expression of inflammation-related programs, which guide the construction of an inflammatory microenvironment, whereas in the extrinsic pathway, inflammatory conditions promote cancer development (e.g., colitis-associated cancer (CAC) of the intestine; Colotta et al., 2009). NF-κB is one of the key orchestrators of cancer-related inflammation (Ben-Neriah and Karin, 2011).

TIR8/SIGIRR was therefore a candidate player potentially involved in cancer-related inflammation and was first studied in a model of CAC, a colorectal disease that arises in patients suffering from chronic IBD, in particular Ulcerative Colitis. In the model of CAC induced by the pro-carcinogen Azoxymethane (AOM), followed by exposure to DSS, which causes chronic inflammation, Tir8/Sigirr-deficient mice exhibited increased susceptibility to intestinal carcinogenesis, in terms of number, size, and severity of lesions, in agreement with the increased susceptibility to inflammation (Garlanda et al., 2007b; Xiao et al., 2007; Figure 3; Table 2). Increased carcinogenesis was associated with increased permeability, local production of pro-inflammatory mediators, such as prostaglandin E_2_, inflammatory cytokines and chemokines, and expression of genes involved in cell survival and proliferation (Bcl-xL and Cyclin D1) downstream of NF-κB. Gut epithelial cells played a pivotal role in mediating the regulatory functions of TIR8/SIGIRR, since TIR8/SIGIRR overexpression in gut epithelium rescued Tir8/Sigirr-deficient mice from developing severe CAC (Xiao et al., 2007). Studies with mice deficient of TLRs, ILRs, MyD88, or Tir8/Sigirr suggest that a fine balance of pro- and anti-inflammatory signals induced by commensal bacteria through TLRs- or ILRs-signaling is necessary for the homeostatic regulation of colon epithelium proliferation and apoptosis as well as for inflammatory responses, mechanisms of repair, and colitis-associated tumorigenesis (Rakoff-Nahoum et al., 2004; Araki et al., 2005; Salcedo et al., 2010). In the AOM-DSS CAC model, mucosal damage induced by DSS causes exposure to commensal microbiota and enhanced migration of bacteria to the mesenteric lymph nodes (Vaishnava et al., 2008). Thus, in this model, Tir8/Sigirr plays a protective role probably by modulating the levels of TLRs signaling in the epithelial cells directly through its interaction with the TLRs that are activated by commensal bacteria. However, the control on pathways activated by TLRs signaling and involving ILRs can not be excluded. In addition, mediators downstream of NF-κB, such as IL-6, which has been shown to promote cancer growth in inflammation-associated cancer models through STAT3 activation, and chemokines, which promote leukocyte recruitment and angiogenesis in gastrointestinal neoplasia (Karin, 2006), were increased in Tir8/Sigirr-deficient mice treated with AOM and DSS.

The role of Tir8/Sigirr was recently investigated in the Apc^min/+^ model, a spontaneous intestinal cancer model mimicking the Familial Adenomatous Polyposis syndrome, where loss of heterozygosity (LOH) of the tumor suppressor Apc is the exclusive genetic alteration leading to the tumor initiation in the Apc^min/+^ mouse (Yamada et al., 2002). Akt-mTOR signaling is a critical pathway driving tumor initiation in the Apc^min/+^ mouse, since it promotes cell cycle progression through posttranscriptional control of the key cell cycle regulators, such as Cyclin D1, cyclin E, and c-Mycand LOH of Apc (Aoki et al., 2003). Xiao et al. (2010) found that Tir8/Sigirr-deficiency in the Apc^min/+^ mouse led to increased microadenoma formation, resulting in spontaneous colonic polyposis. Tir8/Sigirr-deficiency was associated with increased Akt-mTOR signaling and tumor initiation through its impact on cell proliferation and LOH of Apc in epithelium. This phenotype was dependent on the presence of commensal bacteria in the gut, implicating a critical role of TLRs signaling in colonic tumorigenesis in Apc^min/+^ mice; however, hyperactivation of mTOR was also observed upon stimulation of epithelial cells with IL-1. Thus, this study suggests that Tir8/Sigirr is a tumor suppressor that controls colonic tumorigenesis by inhibiting IL-1- and TLRs-induced mTOR-mediated cell cycle progression and consequent genetic instability (Xiao et al., 2010).

## Role of TIR8/SIGIRR in Chronic Lymphocytic Leukemia

Chronic lymphocytic leukemia (CLL) is the most frequent leukemia in the western world and accounts for about 40% of all leukemias in adults over the age of 65 years. CLL is characterized by the accumulation of CD5^+^ monoclonal B-cells in primary and secondary lymphoid tissues. Over time and because of unknown molecular events CLL may progress into an aggressive form characterized by a prolymphocytoid transformation; small cells are gradually replaced by clonally related, larger elements (prolymphocytes). Genetic defects are involved in the pathogenesis of the disease including repeated mutations in the MyD88, XPO1, and NOTCH1 genes (Puente et al., 2011; Rossi et al., 2012). Furthermore, stimuli originating from the microenvironment appear to contribute to the selection and expansion of the malignant clone (Ghia and Caligaris-Cappio, 2006) and CLL is emerging as a prototype of cancers where both genetic and micro environmental factors concur to the development, expansion and progression of the disease (Muzio et al., 2009a,b). Both normal and leukemic human B-cells express detectable levels of *TIR8/SIGIRR* mRNA (Muzio et al., 2009b); however, by PCR array analysis, malignant B-cells appeared to have low levels of mRNA (Arvaniti et al., 2011). Nevertheless, since protein levels of TIR8/SIGIRR may be differentially regulated (Veliz Rodriguez et al., 2012), it will be important to analyze TIR8/SIGIRR expression on the cell surface of normal and leukemic B-cells. As regarding to mouse models, an analysis was performed in TCL1 transgenic animals, a well characterized mouse model of CLL (Bichi et al., 2002). Both total and CD19^+^ enriched splenocytes expressed detectable *Tir8/Sigirr* mRNA levels that were significantly lower in CD19^+^ cells from TCL1 transgenic mice. Moreover, peritoneal B1 cells (which are marked by CD5 expression) expressed lower levels of *Tir8/Sigirr* mRNA compared with splenic B-cells suggesting that an intrinsic program of CD5^+^ B-cells may regulate *Tir8/Sigirr* expression (Bertilaccio et al., 2011).

To address the functional involvement of TIR8/SIGIRR in CLL, *Tir8/Sigirr* was genetically inactivated in the TCL1 transgenic mouse model (Bertilaccio et al., 2011). The absence of Tir8/Sigirr did not modify normal B cell populations in wild type mice. In contrast, lack of Tir8/Sigirr in TCL1 transgenic mice accelerated appearance of monoclonal B-cell expansions and mouse life span was shortened. The morphology and phenotype of the mouse leukemic expansions reproduced the progression of human CLL into an aggressive phase characterized by the appearance of prolymphocytes (Bertilaccio et al., 2011; Figure 3; Table 2). Overall, these observations suggest an inhibitory role of TIR8/SIGIRR in CLL onset and progression. However, it is not clear whether TIR8/SIGIRR exerts its activity directly onto the malignant clone or indirectly through the tumor microenvironment, and which molecular mechanisms are involved. Indeed, different TLRs ligands can lead to either proliferation (TLR2, TLR4, TLR9; Tarnani et al., 2010), apoptosis (TLR3 and TLR9; Liang et al., 2010), or chemoresistance (TLR7 and TLR8; Cherfils-Vicini et al., 2010) of different cancer cells (Rakoff-Nahoum and Medzhitov, 2009). Examples of endogenous TLRs or ILRs ligands, which could constitutively activate cognate membrane receptors in the malignant clone or cells of the tumor microenvironment in the absence of TIR8/SIGIRR, are ILRs family cytokines such as IL-1, IL-18, IL-33, and TLRs ligands including PAMPs derived from intestinal microflora and/or DAMPs containing autoantigens. Thus, this study shows that unabated TLRs and/or ILRs stimulation is functionally involved in the development and progression of CLL, and that TIR8/SIGIRR plays a non-redundant role in controlling this process.

## Concluding Remarks

Interleukin-1R like receptors and TLRs are key inflammatory receptors involved in inflammation and immunity recognizing IL-1 family ligands released in several conditions including sterile inflammation and cell death, or PAMPs and DAMPs, respectively. Available information is consistent with the view that TIR8/SIGIRR is a conserved and broadly expressed negative regulator of inflammation, tissue damage, autoimmunity, and cancer (Figure 3; Table 2), acting by negatively regulating ILRs- or TLRs-dependent signaling, possibly by interfering with the recruitment of TIR domain containing signaling molecules (Figure 2). Although TIR8/SIGIRR is still considered an orphan receptor, IL-36Ra has been proposed as brain-specific TIR8/SIGIRR ligand, and the possibility of other TIR8/SIGIRR recognizing molecule(s) cannot be dismissed.

Members of the ILR family have been involved in T cell polarization and proliferation (Chan et al., 2001; Neighbors et al., 2001; Schmitz et al., 2005; Acosta-Rodriguez et al., 2007) and TIR8/SIGIRR has emerged as a negative regulator affecting Th1, Th2, and Th17 differentiation, which play a major role in autoimmunity and sterile inflammation (Garlanda et al., 2004, 2007b; Xiao et al., 2007; Bozza et al., 2008; Bulek et al., 2009).

Thus, strong genetic evidence in mice is consistent with a non-redundant regulatory function of TIR8/SIGIRR in pathogen-dependent as well as sterile inflammation, innate immunity, and polarized adaptive responses. Data supporting its relevance to human disease are still scarce, however, preliminary genetic evidence in humans and gene expression studies support the hypothesis that TIR8/SIGIRR could play a regulatory function also in human inflammatory conditions.

## Conflict of Interest Statement

The authors declare that the research was conducted in the absence of any commercial or financial relationships that could be construed as a potential conflict of interest.
